# Transcranial photobiomodulation and thermal stimulation induce distinct topographies of EEG alpha and beta power changes in healthy humans

**DOI:** 10.1038/s41598-021-97987-w

**Published:** 2021-09-23

**Authors:** Xinlong Wang, Hashini Wanniarachchi, Anqi Wu, F. Gonzalez-Lima, Hanli Liu

**Affiliations:** 1 Department of Bioengineering, University of Texas at Arlington, Arlington, TX USA; 2 Department of Psychology and Institute for Neuroscience, University of Texas at Austin, Austin, TX USA

**Keywords:** Neuroscience, Engineering, Optics and photonics

## Abstract

Our recent study demonstrated that prefrontal transcranial photobiomodulation (tPBM) with 1064-nm laser enables significant changes in EEG rhythms, but these changes might result from the laser-induced heat rather than tPBM. This study hypothesized that tPBM-induced and heat-induced alterations in EEG power topography were significantly distinct. We performed two sets of measurements from two separate groups of healthy humans under tPBM (n = 46) and thermal stimulation (thermo_stim; n = 11) conditions. Each group participated in the study twice under true and respective sham stimulation with concurrent recordings of 64-channel EEG before, during, and after 8-min tPBM at 1064 nm or thermo_stim with temperature of 33–41 °C, respectively. After data preprocessing, EEG power spectral densities (PSD) per channel per subject were quantified and normalized by respective baseline PSD to remove the power-law effect. At the group level for each group, percent changes of EEG powers per channel were statistically compared between (1) tPBM vs light-stimulation sham, (2) thermo_stim vs heat-stimulation sham, and (3) tPBM vs thermo_stim after sham exclusion at five frequency bands using the non-parametric permutation tests. By performing the false discovery rate correction for multi-channel comparisons, we showed by EEG power change topographies that (1) tPBM significantly increased EEG alpha and beta powers, (2) the thermal stimulation created opposite effects on EEG power topographic patterns, and (3) tPBM and thermal stimulations induced significantly different topographies of changes in EEG alpha and beta power. Overall, this study provided evidence to support our hypothesis, showing that the laser-induced heat on the human forehead is not a mechanistic source causing increases in EEG power during and after tPBM.

## Introduction

Photobiomodulation (PBM), also known as low-level laser therapy (LLLT) in clinical applications, utilizes red to near-infrared (NIR) light to stimulate mitochondrial respiration in a wide range of cells and tissues in the human body^1–4^. Transcranial photobiomodulation (tPBM) is a type of PBM that delivers NIR light/laser to the human brain, which has shown promising outcomes in treating psychiatric and neurological disorders^5^, such as depression and anxiety^6^, and traumatic brain injuries^7,8^. Recent studies have reported that tPBM with a 1064-nm laser can enhance human cognitive performance on a variety of cognitive tasks using sham-controlled experiments^9–13^. Furthermore, we have recently demonstrated that 1064-nm tPBM enabled significant upregulation in concentrations of hemoglobin oxygenation ([HbO]) and oxidized cytochrome-c-oxidase ([CCO]) during and after tPBM on the human right forehead with high reproducibility and robustness^14–16^. These findings supported and validated the hypothesized mechanism of action that tPBM can photo-oxidize CCO, the key mitochondrial enzyme for cellular oxygen metabolism, to boost the metabolic activities of cells^17^, especially neurons^1,18^. In addition, we investigated the thermal impact of tPBM on measured alterations in [HbO] and [CCO], confirming that the heat-induced warm sensation on the forehead would not give rise to the same increases in [HbO] and [CCO] as those seen by tPBM^19^.

There has been much less understanding and observation of electrophysiological responses to tPBM in the human brain. Our recent results revealed that tPBM is effective in enhancing EEG alpha and beta rhythms in the human brain during eyes-opened resting state, measured by 64-channel scalp EEG from healthy humans^20,21^. Similar observations on EEG responses to tPBM were reported by other groups while using different experimental protocols^22–24^. All these studies consistently indicated that tPBM can also modulate neuronal or electrophysiological synchronization and connectivity in the human brain.

It is expected and often experienced that a sizeable optical beam used for tPBM may produce a warm sensation on the subject’s forehead. Since the scalp EEG signal is sensitive to ambient temperature or thermal stimulation of the subject’s head^25^, the warmness created by the light/laser illumination during tPBM can potentially affect or contaminate the net EEG signal induced only by tPBM. Thus, an essential reservation or question on the action mechanism of tPBM would be whether the EEG power or rhythm alteration might result from, at least partially, thermal effects caused by light or laser illumination delivered on the subject’s forehead. To address this important question, we hypothesized in this study that tPBM-induced and heat-induced alterations in EEG power topography at alpha and beta oscillations were significantly distinct or different.

In this study, we investigated both tPBM and thermal stimulation (thermo_stim) conditions using an experimental protocol similar to that in our previous studies^9,14,21,26^. Specifically, we performed two sets of measurements from two separate groups of healthy humans, namely, tPBM group (n = 49) and thermo_stim group (n = 14). Each group participated in the EEG measurements twice under true and respective sham conditions. A 64-channel EEG unit was used for concurrent data collection before, during, and after 8-min active or sham stimulations for each (of tPBM and thermo_stim) measurement under the eyes-closed resting state. Alterations in baseline-normalized EEG power at five frequency bands (delta, theta, alpha, beta, and gamma) and topographical patterns induced by each of tPBM and thermo_stim were then computed and compared. By the end, all these observations provided evidence to support our hypothesis, as progressively presented in the following sub-sections.

## Materials and methods

### Participants

For the sham-controlled tPBM condition, a group of 49 healthy human subjects (30 males, 19 females; 26 ± 8.8 years of age) were enrolled from the local community of the University of Texas at Arlington. For the sham-controlled thermo_stim condition, another group of 14 human subjects (8 males, and 6 females; 29 ± 8.8 years of age) were recruited from the same community. However, 3 subjects from the tPBM measurements and 3 subjects from the thermo_stim measurements were removed from the dataset due to self-reported/observed tiredness and/or sleepiness during the EEG measurements, which resulted in 46 participants for tPBM and 11 participants for thermo_stim in further data analysis. There was no significant age difference between the two experimental groups, nor age difference between two genders in each group (with a two-tailed t-test, p > 0.1). Exclusion criteria of participants: (1) previous diagnosis with a psychiatric disorder, (2) history of neurological disease, (3) history of severe brain injury, (4) history of violent behavior, (5) prior institutionalization or imprisonment, (6) current intake of any psychotropic medicine or drug, (7) history of smoking, (8) excessive alcohol consumption, (9) pregnancy, and (10) previous diagnosis with diabetes as required by the manufacturer of the laser (Cell Gen Therapeutics LLC, Dallas, Texas). All the participants were told to avoid any caffeine beverages 2–3 h before EEG measurement. All experimental procedures were approved by the Institutional Review Board of the University of Texas at Arlington; all methods were performed in accordance with the relevant guidelines and regulations. Informed consent was obtained from each participant prior to all measurements.

### Experimental setup and protocols

**Figure 1:**
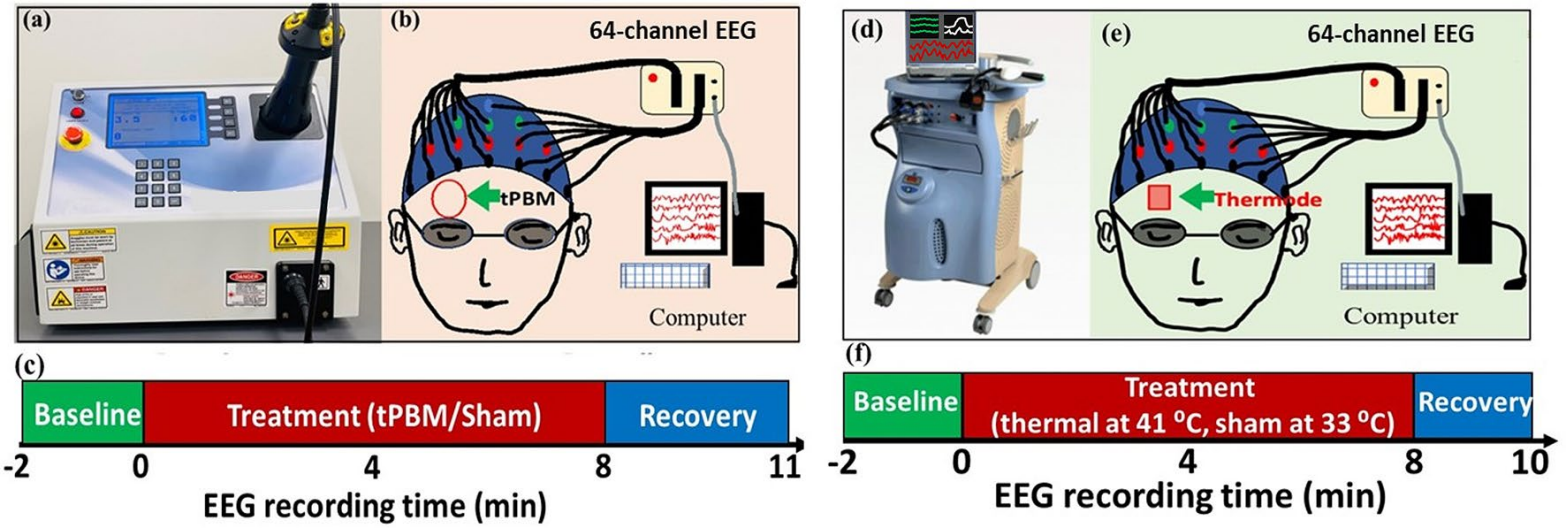
Experimental setups and protocols for concurrent tPBM/EEG and thermo_stim/EEG measurements. (**a**) Photo of FDA-cleared, 1064-nm, continuous-wave laser (Model CG-500). (**b**) Schematic diagram of the experimental setup: 64 electrodes of an EEG device were attached to an international 10–10 standard EEG cap, while the subject was wearing a pair of safety goggles and retained in eye-closed resting state during the sham or active tPBM condition. The laser aperture was pointed at the right forehead of the subject with 2-cm away from the skin. (**c**) The experimental protocol including a 2-min baseline, 8-min stimulation (with a laser power of 3.5 W for tPBM and 0 W for sham), and a 3-min recovery period. (**d**) Picture of the thermal stimulator system (Pathway model with ATS thermode). (**e**) Schematic diagram of the EEG setup used when the subject retained eye-closed during the sham or active thermo_stim condition. The thermode was attached at the right forehead of the subject to produce heat mimicking that created in tPBM. (**f**) The experimental protocol for thermal stimulation: a 2-min baseline, 8-min thermo_stim, and a 2-min recovery period.

As shown in Fig. 1a, a continuous-wave (CW) 1064-nm laser (Model CG-5000 Laser, Cell Gen Therapeutics LLC, Dallas, TX, USA), cleared by the Food and Drug Administration (FDA), was utilized to conduct tPBM and the corresponding sham EEG measurement in this study. This was the same device used in our previous studies^11,19,20,26^. This laser was able to deliver a collimated beam in a diameter of 4.2 cm. We conducted tPBM with a total power of 3.5 W, which led to a power density of ~ 0.25 W/cm^2^ and a total energy dose of 1680 J over 8 min of tPBM (3.5 W × 480 s = 1680 J) on the right forehead, as indicated in Fig. 1b. For the sham measurement, the laser device was on but set to be 0.1 W during the 8-min stimulation time. In addition, a black colored cap was used to block the laser aperture. As a result, the power density under the sham stimulation was further confirmed to be 0 W/cm^2^ by a sensitive power meter (Model 834-R, Newport Corp., Andover, MA, USA) to ensure the complete impediment of laser light. The participants were instructed to maintain their eyes closed with a minimal level of motion during the EEG measurements. Subjects were also directed to give minor hand gestures in response to the experiment operator for verifying that they were not asleep during the measurement. At the end of the measurement, each participant was asked to confirm that he/she was awake without much drowsiness and sleepiness during the entire experimental period.

A Biosemi (64-channel) 10–10 EEG system was employed for data collection. Before each EEG measurement, electrical gel was applied on each electrode to improve the conductivity and signal to noise ratio of the data acquisition. The stimulation protocol (see Fig. 1c) consisted of a 2-min baseline, an 8-min tPBM, and a 3-min recovery period. Under both sham and active tPBM measurement conditions, the laser aperture was pointing at the right forehead near the electrode locations of FP2 and AF8. Both the participant and operator wore protection goggles to prevent any stray laser light from entering their eyes. While the EEG data were acquired at either 256 Hz or 512 Hz, all the 512 Hz data were down sampled to 256 Hz during data preprocessing for consistency. This down-sampling process would not alter the measures of frequency powers^27,28^ since our high end of gamma band was at 70 Hz, 3.66 times less than 256 Hz, obeying the Nyquist sampling theorem.

Designed as a single-blind, cross-over study, each subject took both sham and active tPBM EEG measurements within a period of 1 week, with a minimum of 3 days between the two measurements. The order of the sham or tPBM condition was randomly assigned. After each experiment, every participant was asked whether they perceived any heat sensation on the stimulation site and felt drowsiness during the EEG measurement. A thermal stimulation condition was also designed to explore the impact of tPBM-associated heat sensations on human EEG signals.

Under the thermo_stim condition, a heat stimulator (Pathway model ATS, Pain and Sensory Evaluation system, Medoc Advanced Medical Systems, Israel) was employed (see Fig. 1d) to replicate and induce the thermal stimulation on the human forehead through an ATS mode probe. The ATS thermode can deliver temperatures ranging from 0 to 55 °C with a maximum rate of 8 °C/s for the alteration of temperature. The time-dependent temperature increases on the subject’s forehead induced by active tPBM were remeasured in this study using an infrared clinical thermometer (Medical Head and Ear Thermometer, Metene, England) over several human subjects; the group-averaged results were very consistent with those reported previously^14,19^.

Under the thermo_stim condition, the thermode was placed at the same place as where the tPBM was on the right forehead to simulate/mimic the thermal effect induced by tPBM, as marked in Fig. 1e. The same 64-channel EEG device (as that used under the tPBM condition) was employed to concurrently record electrophysiological responses to the thermal stimulations. Figure 1f shows the experimental protocol: It included a 2-min baseline and an 8-min thermal stimulation, followed by a 2-min recovery period. The temperature of the thermal stimulator remained at 33 °C during the 2-min baselines for both sham and active thermo_stim. For the active stimulation, the temperature of the thermode increased from 33 to 41 °C following the tPBM-equivalent thermal rate^19^ and was maintained at 41 °C during the remaining stimulation period. Then we removed the thermode from the forehead during the 2-min recovery period. For the sham condition, we maintained the thermode’s temperature at 33 °C throughout the 8-min period before removal of the thermode from the forehead while EEG recording lasted the entire 12-min period. Under either true or sham stimulation conditions, subjects were asked to keep their eyes closed throughout the whole measurement time.

### Data analysis

Each collected EEG dataset contained 64-channel time series corresponding to the collected large-scale neural activities at 64 scalp locations. A total of 46 pairs (from 46 subjects) of EEG data under both sham and active tPBM and a total of 11pairs (from 11 subjects) of EEG under both sham and thermo_stim were included for further data processing.

#### Data preprocessing for EEG time series

The EEG data were preprocessed using MATLAB^29,30^ and a MATLAB-based, open-source software package^31^, EEGLAB^32^. First, the acquired 64-channel EEG raw data were bandpass filtered at 1–70 Hz using EEGLAB’s “filtfilt” function. A notch filter was applied to eliminate line noise at 60 Hz. Re-referencing was performed by subtracting the average of voltage signals across all the 64 electrodes from each of the EEG time series. Second, robust principal component analysis (rPCA)^33,34^ was used to identify and remove major signal outliers and artifacts from EEG signals. Then, independent component analysis (ICA)^35,36^ was performed to find noise and artifacts^37,38^, such as eye movements, saccades, and jaw clenches; specifically, we employed “kICA” which implements ICA as the projection of the data that maximizes kurtosis^36^. Then, the two most common noisy components, namely, eye blinks and saccades, were subjectively determined and removed using ICA process for each of the EEG time series.

To better quantify dose-dependent responses to tPBM/sham, we divided each artifact-free time series into four temporal segments: (1) the last 60 s of the 2-min baseline before the onset of tPBM/sham stimulation, (2) the first 4 min (0–4 min) of the tPBM/sham, (3) the second 4 min (4–8 min) of tPBM/sham, and (4) the first 2-min recovery. The preprocessed data were then used to perform further analysis for each of the temporal segments. Likewise, the EEG data under the thermo_stim were preprocessed following the same four temporal segments for both sham and active thermal stimulations.

#### EEG power spectral density and respective percent changes in power

The EEG power spectral density (PSD) of artifact-free time series from each of 64 channels was quantified using Pwelch (with a 4-s window and 75% overlap^39^) in EEGLAB, an open source software package. Our PSD frequency range was selected to be 1–70 Hz, covering delta (1–4 Hz), theta (4–8 Hz), alpha (8–13 Hz), beta (13–30 Hz), and gamma (30–70 Hz) bands. To remove the power-law effect for more sensitive comparison^27^, we calculated percent changes of PSD as power normalization with respect to the last one min of its own baseline within three other temporal segments (i.e., 0–4 min, 4–8 min, and 8–10 min) at five frequency bands, respectively. Because of this normalization step, a bandwidth-averaged percent change in PSD^27^ is also equal to a bandwidth-averaged percent change in power (*ΔmPower in %*) for a given band, as shown below:

1 $$\Delta mPower = \frac{{PSD_{stim} - PSD_{base} }}{{PSD_{base} }} = \frac{{(PSD_{stim} - PSD_{base} ) \times f_{band} }}{{PSD_{base} \times f_{band} }} = \frac{{Power_{stim} - Power_{base} }}{{Power_{base} }} = \frac{{Power_{stim} }}{{Power_{base} }} - 1,$$

where subscripts of “*stim*” and “*base”* represent the stimulation and baseline conditions for the measurement, *f*_*band*_ denotes the bandwidth of a chosen frequency (e.g., 1–4 Hz for delta band), and *PSD*_*stim*_ and *PSD*_*base*_ indicate bandwidth-averaged PSD values. Accordingly, Eq. (1) leads to Eqs. (2a) and (2b) that represent *ΔmPower* in response to active and sham tPBM conditions at each of the 5 frequency bands in each of the three temporal segments, while Eqs. (3a) and (3b) represent *ΔmPower* in response to active and sham thermo_stim conditions for respective spectral bands and time periods:

2a $${\Delta mPower}_{i-tPBM}^{f}=\left(\frac{{P}_{i-tPBM}^{f}}{{P}_{base-tPBM}^{f}}-1\right)100\%,$$

2b $$\Delta mPower_{i - tPBM\_sham}^{f} = \left( {\frac{{P_{i - tPBM\_sham}^{f} }}{{P_{base - tPBM\_sham}^{f} }} - 1} \right)100\% ,$$

3a $${\Delta mPower}_{i-thermo\_stim}^{f}=\left(\frac{{P}_{i-thermo\_stim}^{f}}{{P}_{base-thermo\_stim}^{f}}-1\right)100\%,$$

3b $${\Delta mPower}_{i-thermo\_sham}^{f}=\left(\frac{{P}_{i-thermo\_sham}^{f}}{{P}_{base-thermo\_sham}^{f}}-1\right)100\%,$$

where *P* stands for bandwidth-averaged PSD or Power; *i* represents the three temporal segments; *f* specifies 5 different frequency bands; subscripts of “*base*” indicate the last 1-min baseline segment; subscripts of “*tPBM*,” “*tPBM_sham*,” “*thermo_stim*,” and “*thermo_sham*” denote stimulation types (i.e., tPBM vs. thermal) and conditions (i.e., stimulation vs. sham). These signal processing steps reflected by Eqs. (2a), (2b), (3a), and (3b) were repeated for all 64-channel time series under both tPBM and thermo_stim conditions, respectively, enabling us to form topographies of mean power (or PSD) changes for 5 frequency bands in the three time periods for each human subject.

Next, we calculated sham-subtracted, tPBM-induced percent changes in Δ*mPower* (*SS*_Δ*mPower*) by subtracting Eq. (2b) from Eq. (2a) and arrived at Eq. (4a):

4a $${SS\_\Delta mPower}_{i-tPBM}^{f}=\left(\frac{{P}_{i-tPBM}^{f}}{{P}_{base-tPBM}^{f}}- \frac{{P}_{i-tPBM\_sham}^{f}}{ {P}_{base-tPBM\_sham}^{f}}\right)\times 100\%.$$

Similarly, we obtained sham-subtracted, thermo_stim-induced percent changes in Δ*mPower* by subtracting Eq. (3b) from Eq. (3a) and arrived at Eq. (4b):

4b $${SS\_\Delta mPower}_{i-thermo}^{f}=\left(\frac{{P}_{i-thermo\_stim}^{f}}{{P}_{base-thermo\_stim}^{f}}- \frac{{P}_{i-thermo\_sham}^{f}}{ {P}_{base-thermo\_sham}^{f}}\right)\times 100\%,$$

where all the symbols in Eqs. (4a) and (4b) signify the same as those given in Eqs. (2) and (3). These calculations were carried out easily based on the results from Eqs. (2) and (3) and repeated for all 64 channels for sham-subtracted tPBM and sham-subtracted thermo_stim conditions, respectively, enabling us to form topographies of sham-subtracted mean PSD power changes for 5 frequency bands in the three time periods for each subject.

Since our PSD power calculations were based on ratios of the active/sham period values to the baseline period values, we needed to verify that there were no pre-existing differences between groups or EEG baseline power offsets^40^. Namely, we conducted statistical analysis by (1) first quantifying bandwidth-averaged PSD for each of the five frequency bands during the last 1-min baseline period right before starting tPBM, tPBM_sham, thermo_stim, or thermo_sham, and (2) performing one-way ANOVA to compare these baseline PSDs for each frequency band across the four conditions. Since all the ANOVA results gave rise to p > 0.05 (See Supplementary information A), we ensured that our forthcoming results were not driven or affected by significant baseline differences.

#### Statistical analysis

We performed three sets of statistical analysis to compare significant changes in *ΔmPower* between (1) tPBM and sham effects as determined by Eqs. (2a) and (2b), (2) thermo_stim and sham effects as determined by Eqs. (3a) and (3b), and (3) sham-subtracted tPBM and sham-subtracted thermal effects as quantified by Eqs. (4a) and (4b). First, non-parametric permutation tests^41–43^ were taken at the individual electrode level to determine *p* values for all 64 channels in each of the three sets. Specifically, for the first two sets of analysis, *ΔmPower* values were compared between tPBM (or thermo_stim) and its sham conditions in n = 46 (or n = 11) subjects at each of the five frequency bands and in the three temporal segments. The permutation tests were repeated and returned *p* values for all 64 electrodes at each frequency band. For the third set of analysis, Δ*mPower* values were compared between sham-subtracted tPBM and sham-subtracted thermo_stim, also resulting in 64 *p* values. Next, the 64 *p* values were further statistically tested with the false discovery rate (FDR) correction for multi-electrode comparisons to minimize type I errors among the 64 EEG electrodes at the topography level. A value of α = 0.05 was selected as the FDR threshold for all three comparison sets while α = 0.01 was also used for set 3 to observe improved spatial resolution of significance. Finally, multiple topographies of significant changes in *ΔmPower* were obtained for all three sets of comparisons at 5 frequency bands during the three time periods.

Moreover, due to the unbalanced sample sizes between the tPBM and thermo_stim groups, the effect size (ES) at each electrode was calculated for the comparison between sham-subtracted tPBM and sham-subtracted thermal stimulation. ES is also called Cohen’s *d*, which is defined as the difference between two means divided by the standard or pooled standard deviation of the two groups. In general, d (or ES) = 0.2, 0.5, 0.8, and 1.2 are considered a small, medium, large, and very large effect size, respectively. Given our unbalanced sample size between the tPBM (n = 46) and thermo_stim (n = 11) groups, it was necessary and informative to evaluate ES for statistical relevance of sham-subtracted percent changes in *ΔmPower* between the two groups. This is because “… effect size is independent of sample size. Statistical significance, on the other hand, depends upon both sample size and effect size.”^44^. Also, “The primary product of a research inquiry is one or more measures of effect size, not P values.”^45^.

## Results

We reported results based on three sets of statistical comparisons at the single- and 64-electrode levels. In “Mean percent changes in PSD per electrode”, at the individual-electrode level, we presented percent changes in PSD, mean percent changes in power, *ΔmPower* (%), followed by the statistical significance (i.e., *p* values) on *ΔmPower* values between the active and sham stimulations in each of five frequency bands. In “Topographic changes in *ΔmPower* between active and sham stimulations”, at the 64-electrode level, we reported statistical differences between *ΔmPower* topographies induced by active (i.e., tPBM and thermo_stim) and sham stimulations at the same five frequency bands and during the three temporal periods. In “Distinct topographies of *ΔmPower* between tPBM and thermal stimulations”, we evaluated statistical differences between the sham-subtracted tPBM (i.e., ${SS\_\Delta mPower}_{i-tPBM}^{f}$) versus sham-subtracted thermal (i.e., ${SS\_\Delta mPower}_{i-thermo}^{f}$) effects for the respective frequency bands and time periods.

### Mean percent changes in PSD per electrode

**Figure 2:**
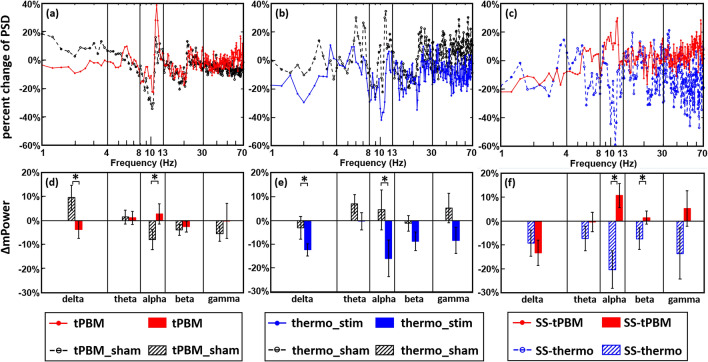
Comparisons of percent changes in PSD at Fp2 during the 4–8 min temporal segment between (**a**) tPBM vs. sham (n = 46), (**b**) thermo_stim vs. sham (n = 11), and (**c**) sham-subtracted (SS) tPBM (n = 46) vs. SS-thermo (n = 11) experimental conditions. The frequency bands are defined as delta (1–4 Hz), theta (4–8 Hz), alpha (8–13 Hz), beta (13–30 Hz), and gamma (30–70 Hz) bands. Furthermore, (**d**)–(**f**) show comparisons of *ΔmPower* (%) at Fp2 averaged over each of the 5 frequency bands during the 4–8 min segment between (**d**) tPBM vs. sham, (**e**) thermo_stim vs. sham, and (**f**) SS-tPBM vs. SS-thermo experimental conditions. “*” indicates statistical significance at p < 0.05 after non-parametric permutation tests. See “Topographic changes in *ΔmPower* between active and sham stimulations” for details.

Since PSD profiles of EEG time series have power-law effects, we took a power normalization approach per channel by calculating percent changes in PSD^27,28^ for the three sets of comparisons and statistical analyses, as shown in Fig. 2. As an example, Fig. 2a plots percent changes of PSD at Fp2 during 4–8 min of tPBM [red trace; based on Eq. (2a)] and sham [black trace; based on Eq. (2b)] stimulations with respect to the last 1-min baseline averaged over all of 46 subjects. With the same plot format, Fig. 2b illustrates percent changes of PSD at Fp2 in response to thermo_stim [blue trace; based on Eq. (3a)] and corresponding sham [black trace; based on Eq. (3b)] conditions (n = 11), while Fig. 2c provides the comparison between percent changes of PSD caused by sham-subtracted (SS) tPBM [red trace; n = 46; based on Eq. (4a)] and SS-thermo_stim [blue trace; n = 11; based on Eq. (4b)]. To be frequency-specific, Fig. 2d–f summarize bandwidth-averaged percent changes in power, after averaging percent changes of PSD within each of the 5 frequency bands during the 4–8 min period of the EEG measurement for the respective three sets of result comparisons. The results shown in Fig. 2 is a representative of quantifications and comparisons of *ΔmPower* for one EEG channel. While the normalized PSD traces seem to be noisy in all three cases (i.e., Fig. 2a–c), quantifications of bandwidth-averaged *ΔmPower* values (in Fig. 2d–f) enabled us to observe group-level differences in *ΔmPower* at Fp2 during 4–8 min between active vs. sham stimulations for both tPBM (n = 46) and thermo_stim (n = 11) groups.

### Topographic changes in *ΔmPower* between active and sham stimulations

**Figure 3:**
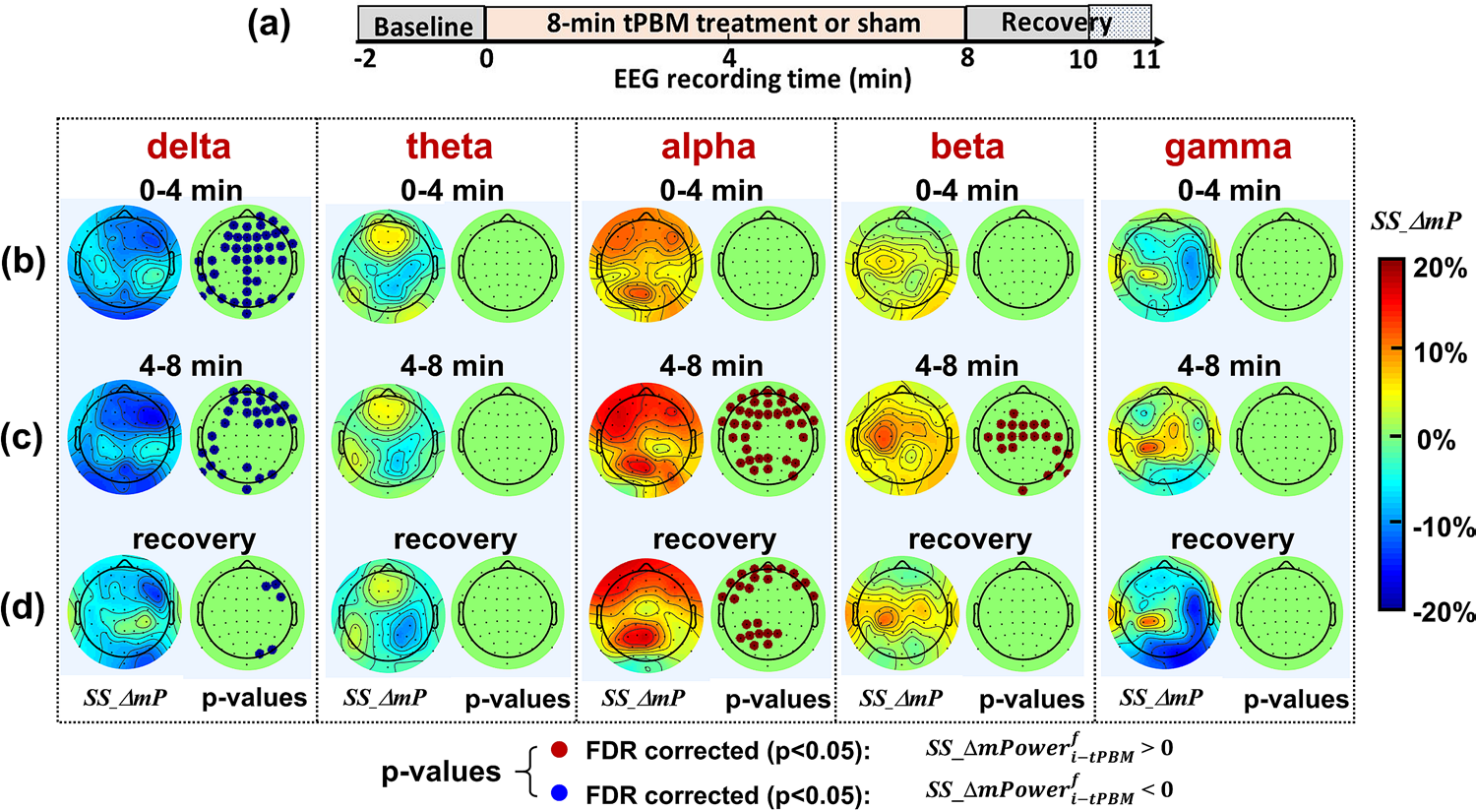
(**a**) Outlines the protocol timing: 2-min baseline, 0–4 min and 4–8 min temporal periods for tPBM/sham, and 8–11 min recovery (only 8–10 min used for analysis). (**b**)–(**d**) show topographies of group-averaged (n = 46), sham-subtracted *ΔmPower* (*SS_ΔmP*) and FDR-corrected p-value maps based on paired permutation tests between SS_ΔmP values under tPBM vs sham conditions during 0–4 min tPBM/sham, 4–8 min tPBM/sham, and 8–10 min recovery, respectively, at five frequency bands. In the *p*-value maps, the red dots correspond to ${SS\_\Delta mPower}_{i-tPBM}^{f}>0 ,$ marking the electrodes where tPBM created significant increases in *ΔmPower* (FDR corrected p < 0.05) compared to the sham condition (see Eq. (4a)). In contrary, the blue dots with ${SS\_\Delta mPower}_{i-tPBM}^{f}<0$ mark the electrodes where tPBM induced significant reduction in *ΔmPower* (FDR corrected p < 0.05) compared to the sham condition.

The processing procedures used to obtain Fig. 2f were repeated for all 64 channels to determine topographic changes in *ΔmPower* under SS-tPBM and SS-thermo experimental conditions, following Eqs. (4a) and (4b), at five frequency bands and during three time segments. For tPBM, Fig. 3 presents five sub-panels for the five frequency bands; each left column in each panel shows three topographies of group-averaged, sham-subtracted *ΔmPower* during (b) 0–4 min tPBM/sham, (c) 4–8 min tPBM/sham, and (d) 8–10 min recovery at five frequency bands. After performing paired permutation tests on each pair of *ΔmPower* values under tPBM and tPBM_sham conditions and obtaining respective *p* values from all 64 channels for respective temporal periods and frequency ranges, we were able to produce p-value maps after the false discovery rate (FDR) correction for respective statistical comparisons, as shown in each sub-panel of Fig. 3 for all five frequency bands during the three temporal segments. Figure 3 shows clearly that *ΔmPower* topographic values in alpha and beta frequencies after sham subtraction were significantly enhanced during the last 4 min of tPBM. Specifically, the sham-excluded enhancement of alpha *ΔmPower* exhibited an anterior–posterior pattern, while the sham-excluded enhancement of beta *ΔmPower* appeared mainly at the central region of the head/brain. Moreover, enhanced alpha *ΔmPower* remained during the recovery period, while the delta *ΔmPower* was significantly reduced during the recovery period.

**Figure 4:**
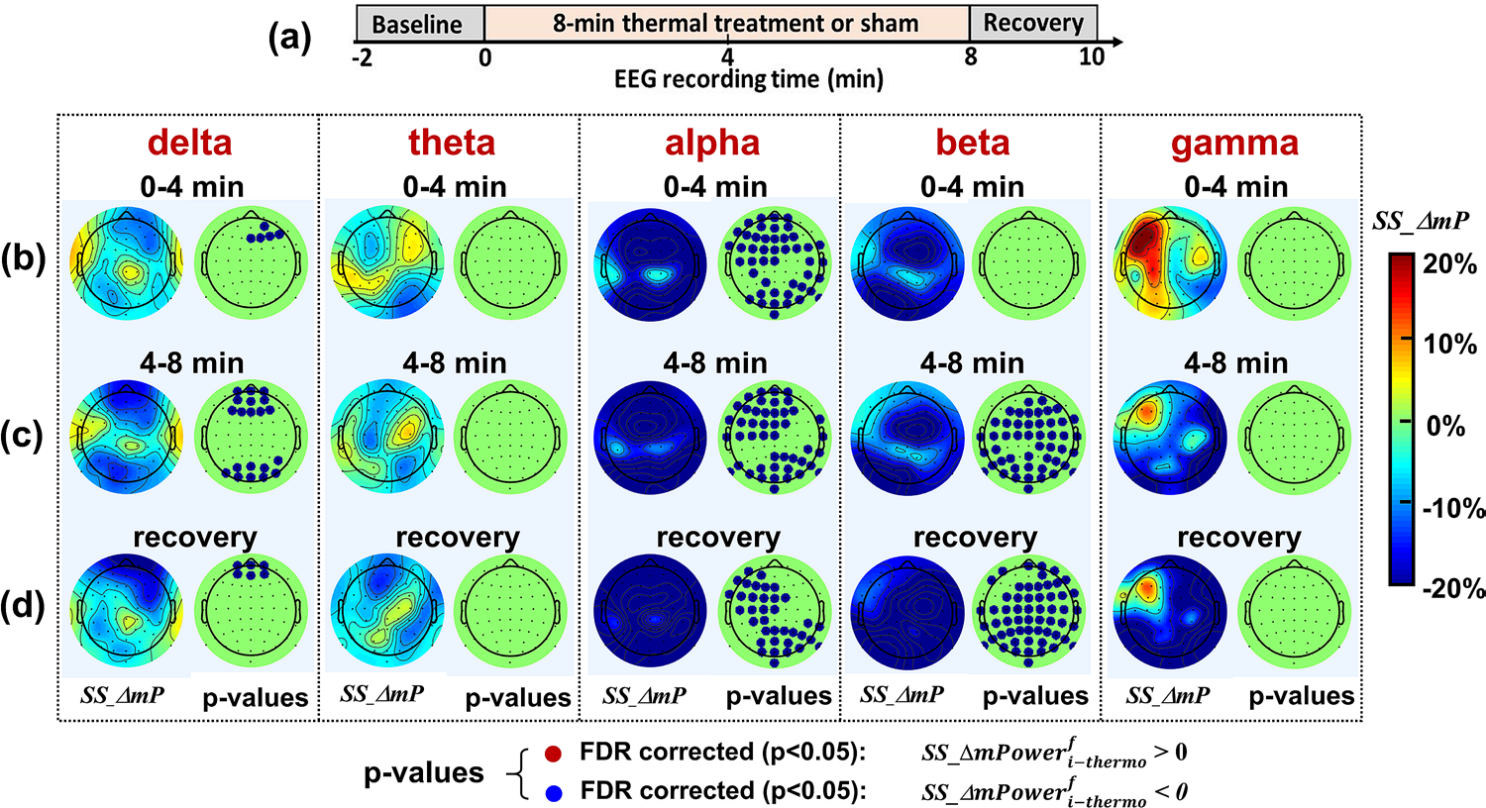
(**a**) Outlines the protocol timing: 2-min baseline, 0–4 min and 4–8 min temporal periods for thermo_stim/sham, and 8–10 min recovery. (**b**)–(**d**) show topographies of group-averaged (n = 11) *SS_ΔmP* and p-value maps with FDR correction based on paired permutation tests between *SS_ΔmP* values under thermo_stim vs sham conditions during 0–4 min thermo_stim/sham, 4–8 min thermo_stim/sham, and 8–10 min recovery, respectively, at five frequency bands. In the *p*-value maps, the blue dots with ${SS\_\Delta mPower}_{i-thermo}^{f}<0$ mark the electrodes where thermo_stim induced significant reduction in *ΔmPower* (FDR corrected p < 0.05) compared to the sham condition. No red dots are shown in any of the topographies at three temporal segments and 5 frequency bands.

Following Eq. (4b) and the same data presentation style as that in Fig. 3, Fig. 4 illustrates the baseline-normalized, group-averaged (n = 11), sham-subtracted topographies of *ΔmPower* (%) by thermo_stim at all five frequency bands. This figure clearly demonstrates that sham-excluded *ΔmPower* in alpha and beta frequencies were significantly reduced, particularly during the last half period of thermal stimulation relative to the sham condition, which is clearly opposite to the patterns seen in the tPBM case (see Fig. 3). Specifically, an anterior–posterior reduction took place in the sham-subtracted alpha *ΔmPower,* while global decreases occurred in the sham-subtracted beta *ΔmPower*. The fact that no red dots are shown in any of the topographies at any temporal segment and frequency band implies that thermo_stim would not generate any increase in EEG powers across the whole head and major frequency bands over an 8-min heating/thermal-stimulation time as compared to those of sham stimulations. Furthermore, sham-subtracted theta and gamma bands did not show any significant changes in *ΔmPower* under thermo_stim.

### Distinct topographies of *ΔmPower* between tPBM and thermal stimulations

**Figure 5:**
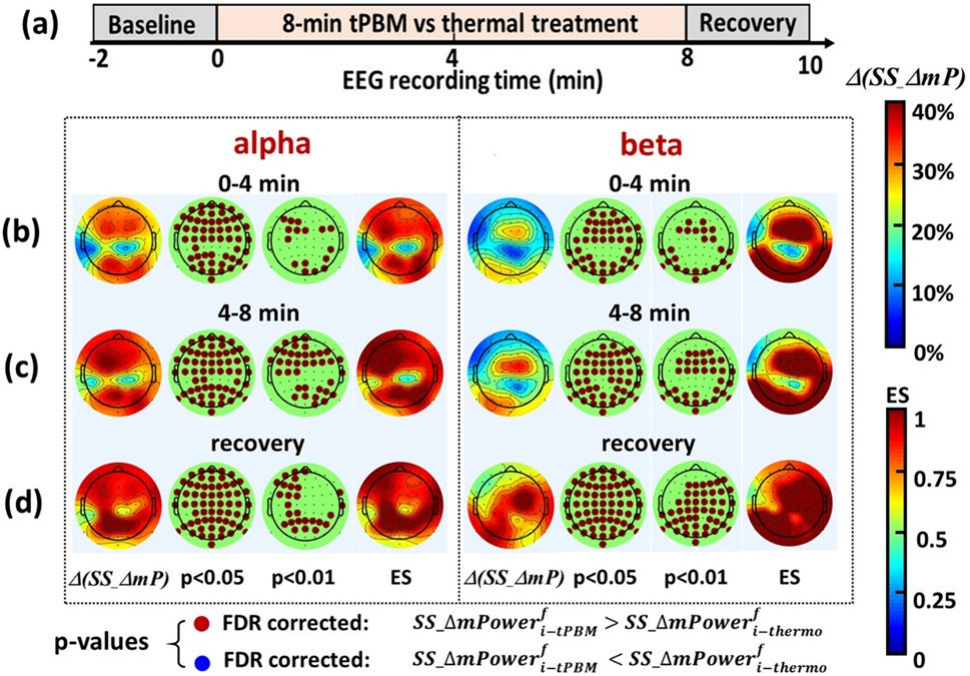
(**a**) Outlines the protocol timing: 2-min baseline, 0–4 min and 4–8 min temporal periods for tPBM/thermo_stim, and 8–10 min recovery. (**b**)–(**d**) show group-averaged differential topographies of *Δ(SS_ΔmP)* (see text for definition) between tPBM and thermo_stim conditions, p-value maps after FDR correction at significance levels of 0.05 and 0.01 based on two-sample permutation tests, and effect size (ES) maps between SS-thermo and SS-tPBM conditions during 0–4 min tPBM/thermo_stim, 4–8 min tPBM/thermo_stim, and 8–10 min recovery, respectively, at alpha and beta frequency bands. In the p-value maps, the red dots denote the electrodes where tPBM created significant increases in sham-excluded *ΔmPower* compared to those with thermal stimulation. No blue dots are shown in any of the p-value topographies at three temporal segments of alpha and beta bands.

As shown above, Figs. 3 and 4 have quantified group-level topographies of sham-subtracted *ΔmPower* (or *SS_ΔmP*) under tPBM and thermo_stim conditions, respectively, for five frequency bands and three time periods. Next, to observe topographical differences of *SS-*Δ*mP* between the two stimulation conditions, we calculated group-averaged differential topographies between them, as defined by *Δ(SS_ΔmP)* = ${SS\_\Delta mPower}_{i-tPBM}^{f}-{SS\_\Delta mPower}_{i-thermo}^{f}$, and shown by the left-most column of each of the two sub-panels for alpha and beta bands in Fig. 5. Furthermore, we conducted two-sample permutation tests between corresponding topographies of *SS_ΔmP* given in Figs. 3 and 4 at all five frequency bands and time periods. This analysis resulted in the two middle columns of each sub-panel in Fig. 5, illustrating the significant difference of the baseline-normalized, sham-subtracted spatial distributions of *EEG* alpha and beta power changes between tPBM and thermal stimulations (i.e., Eqs. (4a)–(4b)) during (b) 0–4 min tPBM/sham, (c) 4–8 min tPBM/sham, and (d) 2-min recovery. Specifically, Fig. 5 includes two sets of p-value maps at α = 0.05 and 0.01 significance levels after FDR corrections, as well as topographies of effect sizes (ES) between SS-tPBM and SS-thermo_stim effects. Since no significant differences in *SS_ΔmP* values were found between tPBM and thermo_stim conditions at delta, theta, and gamma bands, the respective topographies are given only in Supplementary Information B.

As seen in Figs. 3 and 4, thermal stimulation created opposite effects on sham-subtracted *ΔmPower* or *SS_ΔmP* spatial distributions in alpha and beta bands with respect to those by tPBM. This opposite effect gave rise to larger differential changes (i.e., *ΔSS_ΔmP*) between the two stimulation conditions, as further supported by large effect sizes (ES > 0.8) shown also in Fig. 5. Since ES is independent of sample size^44,45^, Fig. 5 illustrated that tPBM-induced *ΔmPower* changes were significantly higher than those by thermo_stim in the anterior–posterior regions for the alpha band and in the central-posterior regions for the beta band during the 8-min stimulation and 2-min post-stimulation period.

## Discussion

In this study, we recorded scalp EEG before, during, and after tPBM/sham from a group of 49 human subjects and thermo_stim/sham from another group of 14 human subjects under the eyes-closed resting state. Percent changes in EEG powers with respect to the baseline were compared between the active and its sham measurements for each of the two groups. The sham-subtracted *ΔmPower* topographies from the tPBM group (n = 46) were compared with the sham-subtracted *ΔmPower* topographies from the thermo_stim group (n = 11). In this way, we rigorously (1) characterized the sham-excluded changes in brain EEG powers under tPBM and thermo_stim conditions, respectively, and (2) presented the evidence that the thermal stimulation did not generate the same alpha and beta power changes as those by tPBM.

### tPBM-induced alterations in EEG *ΔmPower* at alpha, beta, and delta bands

Figure 3 illustrates that tPBM significantly enhanced *ΔmPower* values during the last half period of tPBM in the anterior–posterior regions for alpha oscillations and in the central region for beta oscillations under eyes-closed resting state. These results are in good agreements with those taken under eyes-open resting state, as we reported before^15,16^. The alpha power is believed to be related to wakefulness^46^, cognition-related brain functions (memory encoding, attention, and brain network synchronization)^47–49^, and collaboration of thalamocortical and cortico-cortical interactions ^50^. Previous studies have demonstrated that 1064-nm laser enabled significant behavioral improvements in cognitive functions using the same experimental protocol^3,9–11,51^. Putting all these results together, we speculate that improvement of human cognition by tPBM may be associated with alpha power increases and potential stimulation to the anterior–posterior network, which is an executive network that assists rapid instantiation of new tasks by interacting with other control and processing networks^52^.

Several studies have also presented evidence that improved beta waves are a sign of better cognitive capacity^53,54^. In our case, we observed increases of beta *ΔmPower* near the central cortex covering the somatosensory region, especially left side (Fig. 3). However, this enhancement of beta *ΔmPower* would not result from laser heating based on the topographies derived from thermo_stim (Fig. 4). There must exist another action mechanism of tPBM being able to increase beta power, which is beyond the scope of this study. Furthermore, tPBM reduced delta power significantly during the 8-min intervention. But this reduction could result from laser heating since no significant difference in delta power was found between tPBM and thermal effects (as shown in Supplementary Information B).

### Thermo_Stim-induced alterations in EEG *ΔmPower* at alpha and beta bands

Figure 4 shows that the thermal stimulation, following the equivalent temperature rise given by tPBM, induced significant decreases in alpha powers across frontal-parietal regions and in beta powers globally, meaning significant desynchronizations of alpha and beta waves across the scalp. These observations are consistent with previous EEG studies using non-noxious^55^ and noxious thermal stimuli^56,57^, but most of thermal stimulation sites were on peripheral locations^55,57–60^. One study on tonic pain using continuous EEG to predict subjective pain perception observed significant decreases in alpha (7–10 Hz) power during the stimulation and suggested that this decrease was due to an augmented activity of cortico-cortical and thalamocortical feedback loops^60^. There are numerous EEG-based publications to investigate mechanisms of pain, but they are beyond the focus of this study. The key feature drawn from Figs. 3 and 4 was that the topographic patterns of percentage changes in EEG *ΔmPower* induced by thermo_stim were opposite to those by tPBM at alpha and beta bands. These results confirmed unambiguously that the percentage changes in alpha and beta powers by 1064-nm tPBM could not stem from the thermal impact of the laser used in tPBM.

### Significant distinction in *ΔmPower* topography between tPBM and thermal effects

As shown in Fig. 5, tPBM enabled as large as 30% increases with respect to thermo_stim in differential topographies of sham-excluded *ΔmPower* with large effect sizes of > 0.8 in an anterior–posterior pattern (p < 0.01) for the alpha band and in a central-posterior pattern (p < 0.01) at the beta band, particularly during the 4–8 min tPBM and post stimulation period. This finding is in excellent agreement with one of our previous studies, which presented that the thermal effect was independent and opposite to the tPBM impact on cerebral hemodynamic oxygenation (Δ[HbO]) and metabolic oxidation (Δ[CCO]) near the tPBM site^19^. Taking all these observations together emphasized that 1064-nm tPBM created significant distinctions in alterations of alpha *ΔmPower* across frontal-parietal regions and beta *ΔmPower* globally, Δ[HbO] locally, and Δ[CCO] locally, with respect to those by the thermal stimulation. In addition, the laser/thermal stimulation applied in this study has been proven safe, non-painful, and often little perceptible to human subjects at the laser power density of ~ 250 mW/cm^2^ or lower. A study conducted on a rabbit brain using CW and pulsed lasers demonstrated that the heat generated by a laser with less than 750 mW/cm^2^ does not cause tissue damage^61^.

Since the thermo_stim given in this study generated equivalent heating on the subject’s forehead, it does not rule out heating of the brain as a mechanism. However, a recent study by Dmochowski et al. employed magnetic resonance thermometry to measure brain temperature during 10-min tPBM (*n* = 20) with 808-nm laser and found no significant temperature differences between active and sham stimulation^62^. Another group conducted computer simulations of motor cortex tPBM with 500 mW/cm^2^ at three wavelengths (630 nm, 700 nm, and 810 nm)^63^. They found a temperature increase in the scalp below 0.25 °C and a minimal temperature increase in the gray matter less than 0.04 °C at 810 nm. Similar heating was found for 630 nm and 700 nm used for tPBM, so photothermal effects are suggested to be unlikely in the brain tissue^63^. While no photothermal effect on the human brain by transcranial 1064-nm laser at ~ 250 mW/cm^2^ has been reported, it is reasonable to believe that 1064-nm laser would give rise to heating effects similar to other tPBM lasers.

### Frontoparietal network

As shown, our results presented significant enhancement by tPBM in EEG alpha power or synchronization for frontal-parietal oscillations. It is acknowledged^52^ that the frontoparietal network is a flexible hub for cognitive control and “a distinct control network, in part functioning to flexibly interact with and alter other functional brain networks. This network coordination likely occurs in a 4 Hz to 13 Hz θ/α rhythm, both during resting state and task state.” Thus, it is reasonable to speculate that the ability of tPBM to effectively modulate or synchronize alpha and beta oscillations in the frontoparietal network may be closely associated with or serves as the electrophysiological mechanism of action that tPBM is able to significantly improve human cognition observed by our group^3,9,11,12^ and others^4,7,8,24^.

Moreover, according to Ref.^52^, “precision mapping of individual human brains has revealed that the functional topography of the frontoparietal network is variable between individuals, underscoring the notion that group-average studies of the frontoparietal network may be obscuring important typical and atypical features.” This notion explains why the observed spatial distribution of enhanced EEG alpha and beta *ΔmPower* was rather spread across frontal-parietal regions, in addition to a systematic backwards shift of the EEG cap.

### Limitations and future work

This study also had several drawbacks and thus leaves opportunities for future work. First, the two sample sizes for tPBM and thermo_stim experimental conditions were too unbalanced with the thermal group having too fewer participants (n = 11 used for data analysis), which may cause inaccurate or insufficient statistical conclusions. Second, the international 10–10 EEG cap system in this study was not strictly placed on the human head since a clear area with 4 cm in diameter was needed for tPBM light delivery on the right forehead. Thus, the EEG cap was shifted about 1–2 cm backwards, creating a systematic shift of electrode locations given in Figs. 3, 4 and 5 with respect to the standard 64-electrode locations. Third, we used the FDR correction as a relatively simpler approach to perform multi-channel comparisons for the percentage changes of sham-subtracted EEG powers. As for future work, we will recruit more human subjects for the thermal stimulation experiment to confirm the findings of this paper. We will utilize cluster-based permutation tests to analyze multi-channel EEG time series for more rigorous statistical analysis^64^. Also, a 3-dimensional digitizer will be utilized to quantify exact locations of the 64 electrodes on each subject’s head for correction of the EEG power topography. Last, more quantitative analysis on network connectivity and directional information flow will be taken to substantiate our expectation that tPBM indeed modulates the frontoparietal network significantly during and post tPBM.

## Conclusion

This study demonstrated that baseline-normalized, sham-subtracted tPBM with a 1064-nm laser given on the right forehead of healthy human subjects neuromodulated delta, alpha, and beta oscillations in eyes-closed resting state. Moreover, we demonstrated that thermal stimulations would generate opposite percentage changes in alpha and beta oscillation powers with respect to those by tPBM. After careful two-sample permutation tests and FDR correction, we provided evidence to support our hypothesis that tPBM-induced and heat-induced alterations in EEG power topography at alpha and beta oscillations were significantly distinct during the eyes-closed resting state. The observed significant enhancement on alpha and beta powers by tPBM in the anterior–posterior regions may be the underlying electrophysiological mechanism of action to explain why tPBM enables to improve human cognition.

## Supplementary Information

Supplementary Information.
