# Supplementary material for: Transcranial photobiomodulation and thermal stimulation induce distinct topographies of EEG alpha and beta power changes in healthy humans
Source: Sci Rep. 2021 Sep 23;11:18917. doi: 10.1038/s41598-021-97987-w (PMC8460746; doi:10.1038/s41598-021-97987-w)
Supplement: Supplementary file 1 — Supplementary Information. [file 41598_2021_97987_MOESM1_ESM.docx]

**Transcranial photobiomodulation and thermal stimulation induce distinct topographies of EEG alpha and beta power changes in healthy humans**

**Xinlong Wang^1^, Hashini Wanniarachchi^1^, Anqi Wu^1^, F. Gonzalez-Lima^2^, Hanli Liu^1^**

**Supplementary Material A**

Fig. A below shows the comparison of bandwidth-averaged EEG power spectrum densities (PSD) during the last 1-min baseline period right before starting tPBM, tPBM_sham, thermo_stim, or thermo_sham. The error bars indicate the standard error of the mean. One-way ANOVA to compare the PSDs across these four conditions at each frequency band gave rise to p>0.05 for all cases, demonstrating no significant differences in EEG baseline spectral powers among conditions. The reason for large errors was because of individual variability of frequency-dependent EEG powers from subjects of n=46 and n=11 for tPBM and thermo_stim, respectively. This large variation in PSD baselines demonstrated the necessity to normalize each subject’s EEG time series with respect to their own baseline in order to identify the changes caused by tPBM vs. thermal stimulation.


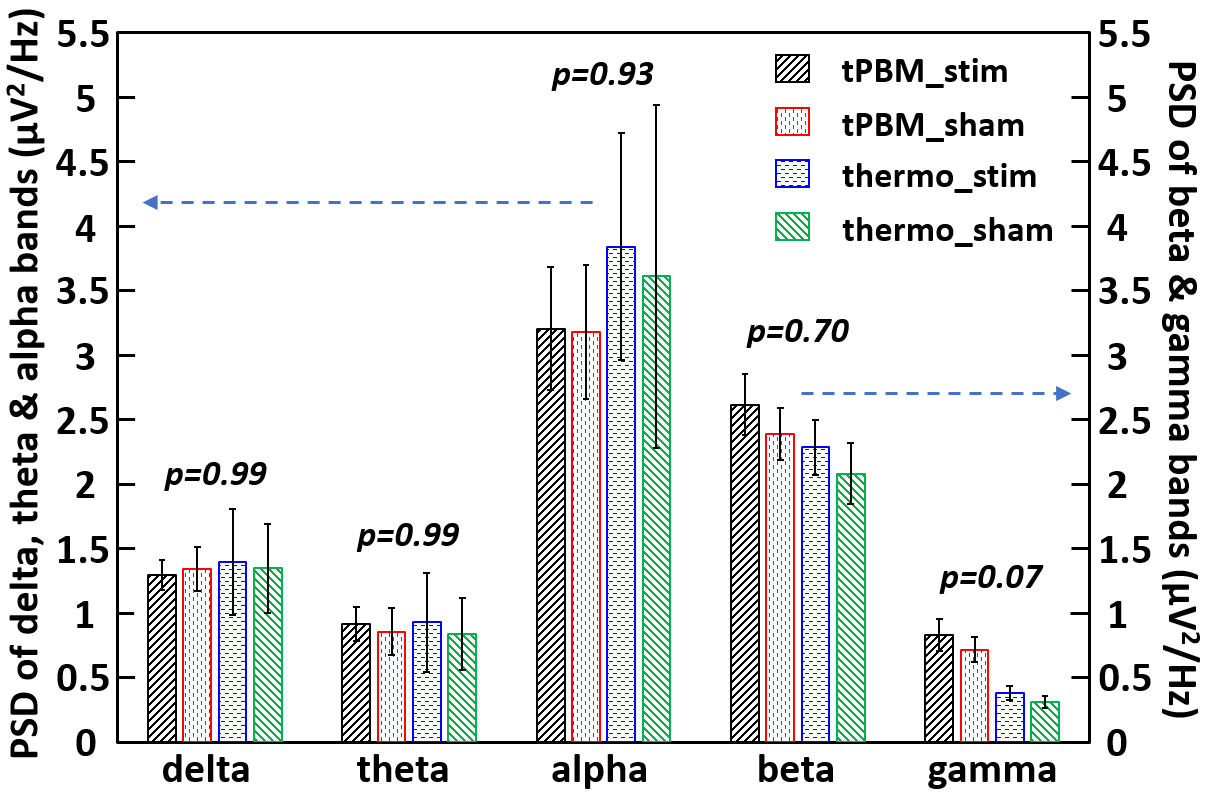


**Fig. A** Comparison of bandwidth-averaged PSD values of delta, theta, alpha, beta, and gamma bands during the last 1-min baseline in tPBM, tPBM_sham, thermo_stim, and thermo_sham experimental conditions. Note that the y-axis marked on the left of figure is for only delta, theta, and alpha bands, while the y-axis marked on the right side is for beta and gamma bands. The error bars are derived from standard errors of the mean. The p values from one-way ANOVA were 0.99, 0.99, 0.93, 0.70, and 0.07 for delta, theta, alpha, beta, and gamma bands, respectively.

**Supplementary Materials B**

**Fig. B** shows (a) the protocol timing: 2-min baseline, 0-4 min and 4-8 min temporal periods for tPBM/thermo_stim, and 8-10 min recovery. (b) – (d) show group-averaged differential topographies of *Δ(SS_ΔmP)* (= ${SS\_mPower}_{i-tPBM}^{f}-{SS\_mPower}_{i-thermo}^{f}$) between tPBM and thermo_stim conditions, p-value maps at significance levels of 0.05 and 0.01 after FDR correction based on two-sample permutation tests, and effect size (ES) maps between SS-thermo and SS-tPBM conditions during 0-4 min tPBM/sham, 4-8 min tPBM/sham, and 8-10 min recovery, respectively, at delta, theta, and gamma bands. These sets of plots show that no significant difference existed in sham-excluded *ΔmPower* between tPBM and thermal stimulations at these frequency bands.

**
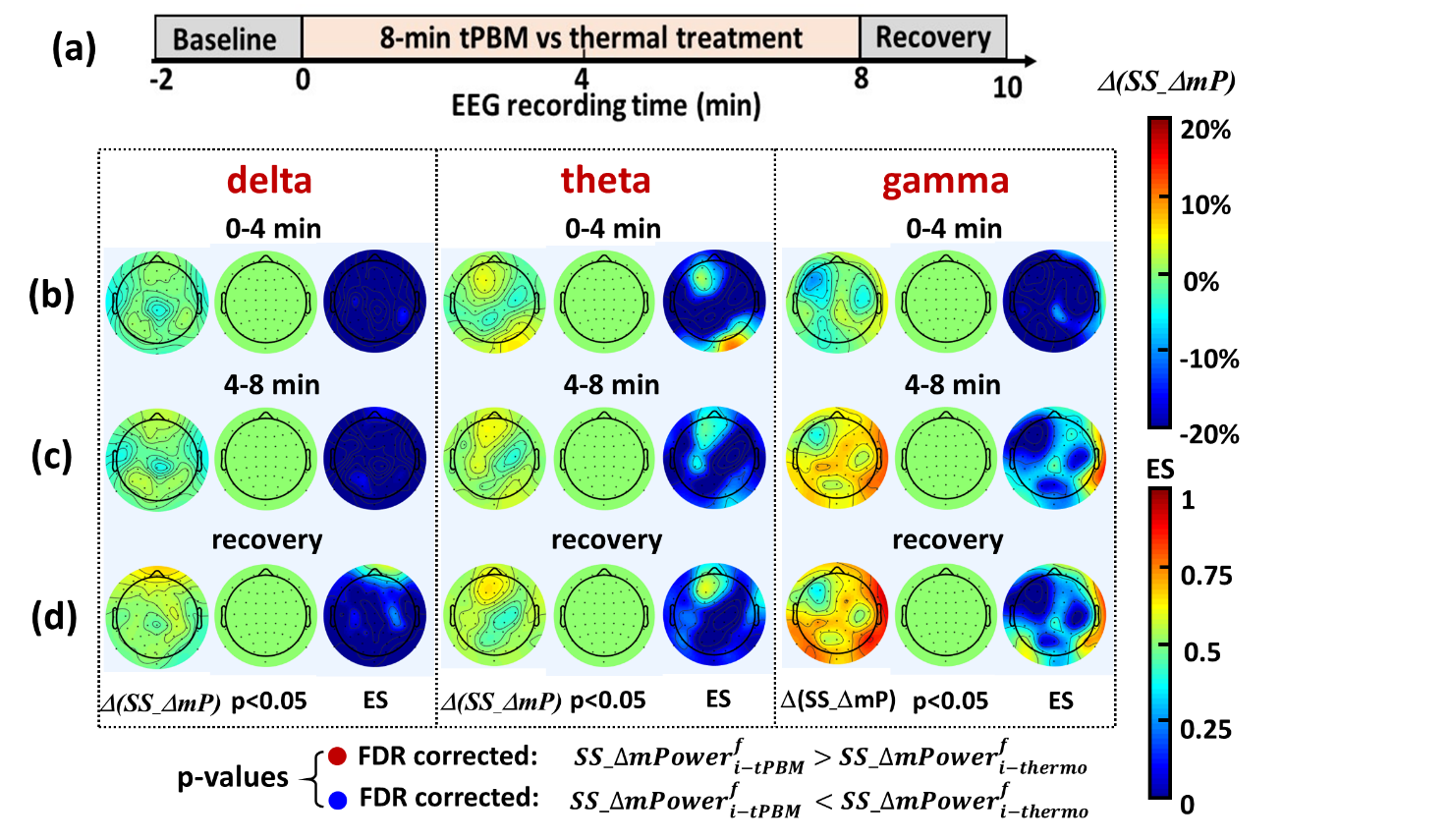
**

**Fig. B** (a) It outlines the protocol timing: 2-min baseline, 0-4 min and 4-8 min temporal periods for tPBM/thermo_stim, and 8-10 min recovery. (b)- (d): In *Δ(**SS_ΔmP)* topographies, the color indicates group-subtracted values of *SS_ΔmP* between tPBM (n=46) and thermo_stim (n=11) experimental conditions. In the p-value maps, there were neither red dots nor blue dots shown in any of the topographies, meaning that SS-tPBM and SS-thermo stimulations did not result in any significant difference in *SS_ΔmP* topographies during all three temporal periods at these three frequency bands, with an FDR-corrected significance level of p<0.05. A large percentage area in each ES topography is shown in blue, implying that the effect sizes in *SS_ΔmP* between SS-tPBM and SS-thermo at most electrode sites were small, in the range of <0.25.
